# The effect of posterior capsule repair upon post-operative hip dislocation following primary total hip arthroplasty

**DOI:** 10.1186/1471-2474-9-29

**Published:** 2008-02-29

**Authors:** Shang-Ju Tsai, Chen-Ti Wang, Ching-Chuan Jiang

**Affiliations:** 1 Department of Orthopedics, Min-Sheng General Hospital, No.168, Chin-Kuo Road, Taoyuan, Taiwan; 2 Department of Orthopaedic Surgery, National Taiwan University Hospital and National Taiwan University College of Medicine, No.7, Chung-Shan South Road, Taipei, Taiwan

## Abstract

**Background:**

Herein, we evaluated, retrospectively, the effect of posterior capsular repair upon postoperative hip dislocation subsequent to total hip arthroplasty (THA) incorporating a posterolateral approach.

**Methods:**

A total of 181 patients undergoing 204 primary non-complicated THA surgical procedures in the period from January 2000 to October 2005 inclusively were included in this study. The patients were separated into two groups by whether the posterior capsular repair had been incorporated in the surgical procedure. For the surgeon did not commence repairing the posterior capsule until July, 2003, all members in the group that did not undergo posterior capsular repair (142 hips from 131 patients) were collected since January, 2000 to July, 2003, while the members in the group that underwent posterior capsular repair (62 hips from 52 patients) were followed since July, 2003, to October, 2005. With a minimum follow-up period of 12 months, we evaluated the early post-operative dislocation rate.

**Results:**

The early postoperative hip-dislocation rate for the group who did not undergo posterior capsular repair appeared to be substantially greater (6.38% versus 0%) than the corresponding figure for the group the members of which underwent posterior capsular repair. In addition, patient demographics and the orientation of acetabular components for the replaced hip joints, as presented in postoperative radiographs, did not differ between the two groups.

**Conclusion:**

Thus, surgeons should include posterior capsular repair as an important step in the surgical procedures of posterolateral approach for all THA in order to reduce the likelihood of early hip dislocation subsequent to THA.

## Background

Hip dislocation following total hip arthroplasty (THA) has been quite a common and bothering complication. The reported incidence of such hip dislocation reaches as high as 9% internationally [1-3]. Certain difficulty as regards achieving a stable hip following THA may result from a variety of causes including the relative spatial positioning of related components following the surgical procedure, the surgical approach, and the level of post-operative compliance in rehabilitation of patients. Over time, a number of different attempts have been made to enhance the stability of the hip joint following hip reconstruction. Specific prosthesis-design modifications such as a reduced head-to-neck diameter ratio and elevated acetabular liners have been shown to reduce the post-operative hip-dislocation rate [4]. Further, abduction braces are frequently used for those patients who, prior to surgery, are considered to reveal a substantial risk of hip dislocation post THA [5]. Patients undergoing THA need to be educated to strenuously avoid some specific postures especially immediately following surgery. Sometimes, however, hip dislocations following THA still occur even after surgeons and patients pay careful attention to these concerns.

The specific surgical approach adopted during THA can often impact upon the postoperative hip-dislocation rate [6]. For example, the use of a direct lateral approach or an anterolateral approach in THA surgery can lead to a lower hip-dislocation rate than the use of a posterolateral approach [6]. The post-operative hip-dislocation rate following a posterolateral approach for THA has been reported to range from 4% to 8% for a general population [3,7,8]. The posterolateral approach for THA, however, is a favoured approach because it features the advantages of good joint exposure, technical simplicity, and, most importantly of all, avoidance of the likelihood of hip-abductor damage [6,9]. The exact reason why the greater post-operative hip-dislocation rate had been associated with the posterolateral approach for THA was not clear at present, but it had been suggested that it might be the result of inadequate posterior capsular support being provided, because for a traditional posterolateral approach, the posterior capsule can either be removed or preserved [3,8,10]. The idea of the repairing of the posterior structures during THA was popularized in 1998 by Pellicci et al who reported reduced post-operative hip-dislocation rates following a posterolateral approach with repairing posterior structures, including short external rotator muscles and posterior capsule, as compared to a posterolateral approach without repairing the posterior structures. The rate for the former ranged from 0% to 0.8% [11]. Encouraged by such good results, our group has developed a technique to allow us to meticulously repair the posterior capsule during THA, a procedure which we have brought into practice at our institution since June, 2004. In order to verify the effect of the posterior capsular repair, for this study, we retrospectively compared the post-operative hip-dislocation rate for patients undergoing THA at our institution prior to and subsequent to introducing this technique of posterior capsular repair as a standard practice during THA surgery.

## Methods

In total, 204 consecutive primary THA procedures from 181 patients were included in this study's test population. All THA procedures were performed by one surgeon through a posterolateral approach. All relevant medical data were collected retrospectively. Study-participating patients were divided into one of two study groups (group I and group II) based upon which surgical procedure was adopted for them. Group I comprised 142 hips from 131 patients for whom the posterior capsule was excised during THA surgery. Group II included 62 hips from 50 patients, for whom careful attention was paid to the preservation of the posterior capsule during THA surgery and meticulous repair of the capsule undertaken at the completion of surgery. Subsequent to THA, the mean early hip-dislocation rate, which was defined as hip dislocation within six months of the completion of surgery [10], was compared for the two groups. All data relating to hip dislocation was derived from our institution's medical records in addition to direct telephone contact being made with study-participating patients.

**Figure 1:**
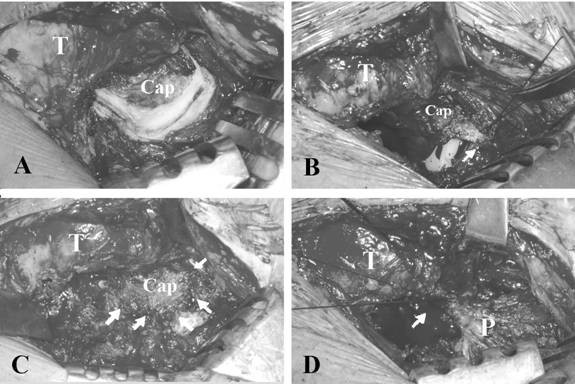
**The intraoperative photographs showed a primary total hip arthroplasty performed through a posterolateral approach with U-shape capsulotomy and capsular repair.** (A) The posterior capsule was exposed following the separation of the piriformis tendon and short external rotator muscles. The U-shaped capsular incision was made along the superior border of the femoral neck, the acetabular rim and then the inferior border of the femoral neck. (B) The capsular flap was carefully protected during the operation and meticulously repaired and relocated back to the original position. The posterior capsule was repaired without the need of capsular plication (arrow). (C) The posterior capsule was repaired. Five stitches with 1-0 Dexon suture were used for this case (arrows). (D) The short external rotator muscles and the piriformis tendon were repaired with the piriformis tendon being repaired first (arrow). T = greater trochanter; Cap= posterior capsule; P= piriformis muscle tendon.

The surgical technique used for the two groups of patients, the standard posterolateral approach, was identical apart from the means of managing the posterior capsule. During this surgical procedure, the short external rotator muscles and piriformis tendon were divided at its insertion site on the trochanter and separated carefully from the posterior capsule. For group-I members, capsulectomy without attempt of capsular repair was performed with a size of that allowing the femoral head to be dislocated out of the acetabulum. For group-II individuals, a U-shaped capsulotomy featuring capsular-base attachment to the proximal femur was performed. The capsular incision was made along the superior border of the femoral neck, the acetabular rim, and then the inferior border of the femoral neck (Fig 1A). A U-shaped capsular flap was then formed with its base attached on the femoral neck. The capsular flap was carefully protected during the operation and meticulously repaired and relocated back to the original position (Fig 1B and Fig 1C). For both groups, the short external rotator muscles and the piriformis tendon were repaired (Fig 1D). Postoperative patient care included patient instruction regarding appropriate training exercises as also generalised patient education relating to their adapting to their having been fitted with a prosthetic device.

For all patients, the orientation of the acetabular cup was carefully evaluated from the postoperative radiographs. Further, standardized anteroposterior radiographs of the pelvis centred over the symphysis pubis were used in order to determine the abduction angle and the true anteversion angle of the acetabular cup. The abduction angle was determined from the angle formed by a line described through both acetabular teardrops and a line drawn through the medial and lateral margin of the acetabular component [12]. The anteversion angle of the acetabular component was measured using a new protractor developed in 2006 [12]. All hip prostheses used for this study were manufactured by Zimmer, Inc (Versys^® ^Fiber Metal Taper stem and Trilogy^® ^acetabular system cup, Zimmer Inc, USA). The diameter of the head of the femoral component used was 28 mm. The bearing surface for the metal acetabular component was a standard non-elevated liner made of high molecular-weight polyethylene. No devices that could possibly affect the post-THA hip-dislocation rate, such as abduction braces, were used for this study.

Inter-group Chi-square testing was used to distinguish, statistically, between study data as regards patient gender distribution and independent-sample T test was used to distinguish between study data as regards patients' follow-up time and age. Fisher's exact probability test was used to compare inter-group hip-dislocation rate and to distinguish any statistically significant difference that may have existed as regards post-THA dislocation rate. One-way ANOVA testing was used to compare results as regards the abduction angles and anteversion angles of the hip acetabular components for each group. Statistical significance was set at a P-value of < 0.05. Institutional review board approval was given in this retrospective study

## Results

In total, 102 females (119 hips) and 82 males (85 hips) participated in this study. Group I was comprised of 71 females (79 hips) and 63 males (63 hips), and group II was comprised of 31 females (40 hips) and 19 males (22 hips). The average age of study participants from group I was 58.55 years (range from 24 to 87, median 61) and that for group-II members was 63.32 years (range from 35 to 90 years, median 66). The preoperative diagnoses of members in group I were primary osteoarthritis in 99 hips, osteonecrosis in 27 hips, post-traumatic osteoarthritis in 10 hips and rheumatic arthritis in 6 hips. In the other hand, the preoperative diagnoses of members in group II were primary osteoarthritis in 44 hips, osteonecrosis in 10 hips, posttraumatic osteoarthritis in 3 hips and rheumatic arthritis in 5 hips. All patients were followed-up for at least 12 months. The average follow-up period for group-I members was 51.6 months (range form 24 to 77) and for group-II individuals, it was 14.8 months (range from 12 to 22). The average follow-up period for all patients was 38.3 months (range from 12 to 77). Summary details relating to average patient age and follow-up period are listed in Table 1. All patients featured at least 12 months of follow-up. As regard to patient age and gender distribution, there was no obvious difference between these two groups. However, inter-group difference was significant for the demographic data of mean follow-up period (Table 1).

**Table 1:** Patient demographics

	**Male (Hips)**	**Female (Hips)**	Age (year)*	Follow-up (month)*
Group-I patients	63 (44.4%)	79 (55.6%)	58.56 ± 14.06	51.6 ± 16.82
Group-II patients	22 (35.5%)	40 (64.5%)	63.32 ± 13.31	14.8 ± 4.47
Tests of inter-group difference	P = 0.303^†^		P = 0.21^§^	P = 0.01^§^

*values are mean ± standard deviation. ^†^Chi-square test. ^§^Independent-sample T test

For group I, post-THA hip dislocations occurred for ten of 142 hips (7.04%), whereas for group II, no dislocation was reported for a total of 62 hips. The preoperative diagnoses of these dislocated hips were primary osteoarthritis in 7 hips and osteonecrosis in 3 hips. All hip dislocations occurred within six months of THA surgery apart from one case, a 38-year-old individual who underwent THA due to right femoral-head osteonecrosis, and who experienced dislocation 24 months subsequent to THA surgery. This dislocation occurred following accidental slipping in the bathroom. As best we were able to determine, all other dislocations occurred within six months of THA surgery and without any apparent traumatic reason. Using the definition of early dislocation as being hip dislocation occurring within six months of THA surgery [10], the early dislocation rate for group-I members was 6.38% (nine out of 142 hips), a figure which was substantially greater than the corresponding figure for group II (Table 2).

**Table 2:** The dislocation rate between groups

	**Dislocation**	**Non-dislocation**
Group I	9 (6.34%)	133 (93.66%)
Group II	0 (0%)	62 (100%)
Tests of inter-group difference	P = 0.035*	

* Fisher's exact probability test.

As regards the hip acetabular components for group I, the average abduction angle was 38.16° (range from 30° to 43°) and the average anteversion angle was 9.86° (range from 4° to 15°), and for group II, the average abduction angle was 37.4° (range from 33° to 41°) and the average anteversion angle was 11.1° (range from 5° to 14°). For those group-I individuals that experienced hip dislocation, the average abduction angle was 37.5° (range from 30° to 40°) and the average anteversion angle was 10.67° (range from 5° to 15°) whereas the corresponding angles for group-I patients that did not experience hip dislocation were, respectively, 38.2° (range from 31° to 43°) and 9.8° (range from 4° to 15°). As regards the relative orientation of acetabular components (including abduction angles and anteversion angles), no difference was significant between the dislocated group-I patients, the non-dislocated group-I patients and group-II patients (Table 3).

**Table 3:** Orientation of acetabular component and the early post-operative hip-dislocation rate (within six months of surgery) for the two groups

		Abduction angle*	Anteversion angle*
Group I	Non-dislocation	38.2° (31°–43°) ±3.485	9.8° (4°–15°) ±2.995
Group I	Dislocation	37.5° (30°–40°) ±4.276	10.6° (5°–15°) ±2.604
Group II	Non-dislocation	37.4° (33°–41°) ±2.652	11.1° (5°–14°) ±2.672
Tests of inter-group difference		P = 0.721^†^	P = 0.332^†^
Group II	Dislocation	No patient	No patient

*values are mean (range) ± standard deviation. ^†^One-way ANOVA test (3 groups).

## Discussion

Herein, we present evidence that posterior capsular repair can decrease the hip-dislocation rate following THA surgery. The influence of posterior-capsular repair being incorporated as part of THA surgery would appear to be very evident from our work, so much so that such a surgical step being incorporated in THA surgery led to a decrease in the early hip-dislocation rate following THA from 6.38% (not incorporated in THA) to 0% (subsequent to its incorporation in THA). This result is analogous to an "all-or-none" study, a result which would suggest level-one evidence for therapy in evidence-based medicine [13].

From a review of the literature, we note that earlier attempts to repair the posterior structure of hip have been previously proposed by various surgeons in order to attempt to decrease the rate of posterior dislocation of the hip following THA using a posterolateral approach [10,11,14-16]. In 1990, Hedley et al. reported on the routine reattachment of the posterior capsule and the short external rotator muscles in one layer to the greater trochanter using multiple sutures [15]. Only two traumatic dislocations were noted in 259 hips for a minimum follow-up of one year. The reported dislocation rate was 0.4%. In 1998, Pellici et al. reported a surgical THA technique wherein the posterior capsule and short external rotator muscles were repaired separately by non-absorbable sutures [11]. They attached these two structures to the greater trochanter but in one single layer. These workers retrospectively compared the incidence of posterior hip dislocation prior to and following their having commenced repairing the posterior capsule in this fashion as a routine part of their THA procedure. From such a review, these authors reported that the incidence of hip dislocation for the group the members of which underwent posterior capsule and short external rotator muscles repair as completed by one of two different senior authors was 0.8% for one surgeon and 0% for the other [11]. In 2001, White et al. developed a technique for creating a posterior capsular flap in which the short external rotator muscles and posterior capsule were not separated, and the posterior capsular flap was repaired and sutured back to the greater trochanter. The incidence of posterior hip dislocation was reported to be 0.7% (three out of 437 hips) for this study [10].

In our study, we went to quite some effort to attempt to minimize the influence of confounding factors in order to overcome the weaknesses of retrospective study design. We used implants manufactured by the same company for all patients. The solitary surgeon used the same surgical approach throughout and repaired the short external rotator muscles and the piriformis tendon for all patients. The only difference between the two groups related to the surgical strategy adopted for managing the posterior capsule. Since mal-positioning of the acetabular components during THA has long been reported to be an important cause of subsequent hip dislocation [17,18], we also incorporated the orientation of the acetabular components of the THA post-operatively for all patients participating in the analysis of this study. We measured the abduction angles and anteversion angles and demonstrated that no difference between group I and group II existed in this regard. In addition, we did not detect any differences in the orientation of acetabular components between dislocated hips and non-dislocated hips from group I. Considering these results, and the fact that the post-operative dislocation rate for group II was much lower than that for group I, it appears reasonable to assume that the strategies for managing the posterior capsule during THA surgery as adopted for group-II patients is an extremely important determinant of postoperative hip-dislocation rate. In other words, posterior capsular repair during THA can dramatically decrease the incidence of postoperative hip dislocation for THA performed via a posterolateral approach.

From our study, the rate of early hip dislocation (within six months of surgery) following THA was 6.34% (nine of 142 hips) for group-I patients, for whom we repaired the short external rotators only. This dislocation rate is similar to that reported in several other studies, for which neither the posterior capsule nor the short external rotator muscles of the patients were repaired during THA surgery [10,11]. We repaired the posterior capsule and short external rotators for group-II patients participating in our study, and the early dislocation rate for this group was 0%. Thus, it appears that during THA surgery, the posterior-capsular repair, but not the repair of the short external rotator muscles, is the critical factor affecting the incidence of postoperative hip dislocation. In 2004, Dixon et al. proposed a method for simple capsulorrhaphy to the gluteus medius tendon without reattachment of the short external rotator to the great trochanter [14]. For this study, only one hip dislocation from 255 hips was noted following a minimum two-year follow-up. Such a result would appear to verify our conclusion further that posterior capsular repair may be an extremely important factor for preventing postoperative hip dislocation subsequent to THA.

The mean follow-up period for group-I patients was 36.8 months longer than was the case for group-II individuals participating in this study. The reason for this is that the surgeon did not commence repairing the posterior capsule until July, 2003, at which time the U-capsulotomy technique was developed, and the posterior capsule repaired during THA surgery for all patients. All members in group I were followed since January, 2000, while the members in group II were followed since July, 2003. Although the mean follow-up time was different for the two groups in our study (P = 0.01), all dislocations apart from one occurred within six months of THA surgery. We propose that the purpose of posterior capsular repair is to decrease the posterior dislocation rate within a short space of time of the completion of THA surgery and prior to the forming of a fibrous pseudocapsule around the hip joint. For our study, all patients from group II were followed-up for at least 12 months, a time period which we would suggest is sufficiently long to allow for the forming of a fibrous pseudocapsule around the hip joint. This thus suggested that it's appropriate to compare the early dislocation rate for the two groups. As such, the difference in follow-up time between the two study groups would not appear to have affected our results.

## Conclusion

In this study, we have demonstrated that the repair of the posterior capsule as a part of THA surgery, but not the repair of the short external rotator muscles, significantly reduced the incidence of posterior hip dislocation following THA surgery using a posterolateral approach. We suggest that surgeons should, in addiction to the repair of the short external rotator muscles, consider posterior capsular repair as an important step in the posterolateral approach to all THA procedures, in order to reduce the likelihood of early hip dislocation subsequent to THA surgery.

## Competing interests

The author(s) declare that they have no competing interests.

## Authors' contributions

SJ carried out the data acquisition, analysis and interpretation of data and drafted the manuscript. CT participated in the design of the study and performed the statistical analysis. CC conceived of the study, and participated in its design and coordination and helped to draft the manuscript. All authors read and approved the final manuscript.

## Pre-publication history

The pre-publication history for this paper can be accessed here:


